# Oral microbiome in human health and diseases

**DOI:** 10.1002/mlf2.12136

**Published:** 2024-09-16

**Authors:** Siqi Tian, Tao Ding, Hui Li

**Affiliations:** ^1^ Department of Immunology and Microbiology, Zhongshan School of Medicine Sun Yat‐Sen University Guangzhou China; ^2^ Key Laboratory of Tropical Diseases Control (Sun Yat‐Sen University) Ministry of Education Guangzhou China; ^3^ Key Laboratory of Human Microbiome and Chronic Diseases (Sun Yat‐sen University) Ministry of Education, China Guangzhou China

**Keywords:** human diseases, microbiome dynamics, microbiome intervention, microecology, oral microbiome

## Abstract

The oral cavity contains the second‐largest microbiota in the human body. The cavity's anatomically and physiologically diverse niches facilitate a wide range of symbiotic bacteria living at distinct oral sites. Consequently, the oral microbiota exhibits site specificity, with diverse species, compositions, and structures influenced by specific aspects of their placement. Variations in oral microbiota structure caused by changes in these influencing factors can impact overall health and lead to the development of diseases—not only in the oral cavity but also in organs distal to the mouth—such as cancer, cardiovascular disease, and respiratory disease. Conversely, diseases can exacerbate the imbalance of the oral microbiota, creating a vicious cycle. Understanding the heterogeneity of both the oral microbiome and individual humans is important for investigating the causal links between the oral microbiome and diseases. Additionally, understanding the intricacies of the oral microbiome's composition and regulatory factors will help identify the potential causes of related diseases and develop interventions to prevent and treat illnesses in this domain. Therefore, turning to the extant research in this field, we systematically review the relationship between oral microbiome dynamics and human diseases.

## INTRODUCTION

Changes in the human oral microbiome have been linked to various diseases, indicating the need to develop a microbiome research platform to analyze this connection in depth. Following the accomplishments of the Human Microbiome Project^1^ and the Human Oral Microbiome Database^2^ in 2007, oral microbiology research entered a new phase^3^. Over 2000 oral reference genomes are now stored in the database^4^. This information has been used to analyze the types and functions of bacteria in the oral cavity, draw a distribution map of oral microbes, and assess the composition of each microbe^5^.

Studies have shown that distinct physical forces and chemical components at different oral sites profoundly influence the composition, quantity, and stability of oral microbiome^6^, ^7^. Researchers have highlighted that disorders and changes in the stability of the oral microbiome are highly likely to lead to various diseases^8^, such as dental caries and Alzheimer's disease. In turn, these diseases can impact the oral microbiota, further exacerbating the instability^1^.

This review identifies the factors that impact the oral microbiome, summarizes diseases caused by oral changes in microbiome composition and structure, and discusses the potential value of preventing and treating illnesses by intervening in the oral microbiome.

## COMPOSITION, DISTRIBUTION, AND HETEROGENEITY OF THE ORAL MICROBIOME

### General composition of the oral microbiome

The oral cavity of a healthy individual is inhabited by multiple bacterial species—mainly those belonging to the phyla *Firmicutes*, *Actinobacteria*, *Bacteroidetes*, *Fusobacteria*, *Proteobacteria*, and *Spirochetes* ^4^. Most oral bacteria are either anaerobic or facultatively anaerobic and can thrive in the absence or presence of oxygen. Among these bacteria, the most prevalent and abundant genus is *Streptococcus*, a member of *Firmicutes*, which represents approximately 20% of all oral bacteria^9^.

### Site specificity of oral microbiome distribution

**Figure 1:**
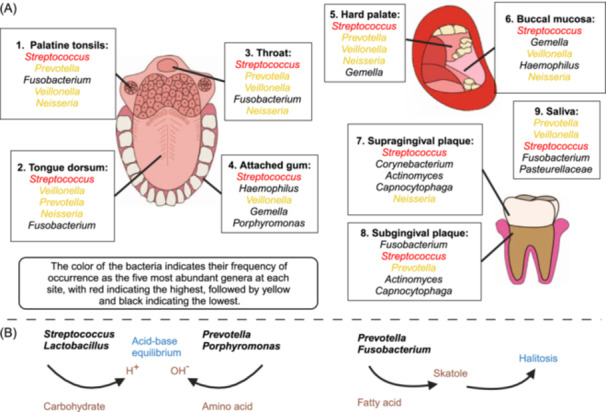
Structure and function of the microbiome at nine microbiome loci in the oral cavity along with corresponding diseases. (A) Different chemical compositions and physical forces form nine specific oral cavity sites. Each harbors a specific microbiome composition^10^. The genera of the top five bacterial species at each site are listed^11^. The color of each bacteria indicates the frequency of its occurrence. (B) Bacterial metabolism produces various metabolites that exert both beneficial and harmful effects on the oral cavity. Some can produce amino acids, while others produce acidic substances to neutralize salivary pH and maintain the steady state of the oral environment. In contrast, *Prevotella* and *Fusobacterium* can also produce skatole, which causes halitosis^12^.

Several research teams have investigated the distribution of oral microbiota across different oral sites. Welch et al. classified the oral cavity into nine distinct areas based on their physical and chemical properties: the specialized epithelium (tonsils and back of the tongue), throat and attached gum, hard palate and buccal mucosa, tooth surface (supragingival and subgingival), and saliva^10^. Each site has its own unique bacterial composition^11^ (Figure 1A). But the biofilms and planktonic bacteria are not static. The top of the attached bacteria can detach, enter the saliva, and be transported to new colonization sites.

These site‐specific differences in microbial community composition have been attributed to various physical and chemical factors, such as surface topology, epithelial tissue structure, and oxygen availability. For instance, oxygen‐deprived subgingival surfaces predominantly harbor anaerobic bacteria, whereas relatively oxygen‐rich supragingival areas support more aerobic species. The supragingival plaque mainly comprises facultative anaerobic Gram‐positive bacteria, such as *Streptococcus*. In contrast, the subgingival plaque is enriched with anaerobic Gram‐negative bacteria, such as *Fusobacterium nucleatum*, *Prevotella intermedia*, *Actinomycetes actinomycetemcomitans*, *Tannerella forsythia*, and *Porphyromonas gingivalis* ^13^. In addition, the specialized epithelia of the dorsal tongue and tonsils harbor unique bacterial communities owing to their distinctive structures, such as crypts and taste buds.

### Metabolism of oral microbiota

The diverse assemblage of microorganisms in the oral cavity changes under external influences. A healthy structure may be maintained, or pathogenic and opportunistic bacteria may propagate, leading to diseases. For instance, planktonic bacteria in saliva are more likely to be affected by the chemical composition of saliva, whereas biofilms are more likely to be affected by physical forces. A biofilm is composed of many different bacterial species attached to the tissue surface. Because of their relatively compact structure compared with planktonic bacteria, biofilms have the ability to resist certain external influences. Foreign pathogenic bacteria must switch from a planktonic to a biofilm‐bound state to invade the human body. Compared with biofilms, planktonic microbial cells interact less, weakening synergistic and antagonistic biochemical interactions^14^. Both in vivo^15^ and in vitro^16^ experiments have shown that biofilms have a stronger tolerance than planktonic bacteria because of the different degrees of binding tightness they create between the bacteria and the surrounding microenvironment. Bacteria involved in biofilm composition can also make direct physical contact to enable signaling or use diffusible chemicals and electrical signaling waves. In contrast, planktonic bacteria communicate mainly through chemicals in saliva^17^.

The metabolism of the oral microbiota is characterized by various forms that play a crucial role in the communication and interaction between the bacteria and the host, as well as among different bacteria species. Bacteria can use metabolites of other genera and form symbiotic relationships. For example, *Streptococcus* spp. produces lactic acid, acetic acid, and hydrogen peroxide, which promote the growth of *Corynebacterium* and produce caries. In contrast, *Staphylococcus* and *Streptococcus* spp.^18^ can use nitrate to produce nitrite and then nitric oxide (NO)^19^, ^20^, inhibiting the growth of *Lactobacillus*, which is another caries‐related pathogen. *Streptococcus* spp. can also produce arginine deiminase to inhibit the colonization of tissue surfaces by *A. actinomycetemcomitans* ^21^. Additionally, it can reduce the production of harmful H_2_O_2_ by *F. nucleatum* to ameliorate oral mucositis^22^.

Bacterial metabolites regulate the internal environment of the oral cavity. *Streptococcus, Actinomyces*, and *Lactobacillus* can use carbohydrates to produce acidic substances that reduce saliva's pH. Conversely, *Prevotella* and *Porphyromonas* can decompose proteins to produce amino acids and alkaline ammonia to neutralize the pH of saliva and maintain a steady state in the oral environment. Moreover, *Prevotella* can metabolize fatty acids, sulfur compounds, indoles, and ammonia to produce various chemicals that cause halitosis, including skatole^12^ (Figure 1B).

### Heterogeneity of human oral microbiome

The factors that influence the oral microbiome in varying ways among different people include age, race, sex, diet, geographical location, and oral hygiene habits. Some of these factors, such as age, can be easily analyzed quantitatively. However, other factors, such as geography and ethnicity, may be more challenging and are thus often discussed only in terms of their influence on diet.

The microbiota typically requires several years to mature after birth and take on its adult form. Studies have shown that a baby's oral microbiota derives not from the amniotic fluid but from food consumed after birth^23^. After puberty, oral bacteria in the same area undergo minimal changes^24^. However, during pregnancy, the composition and abundance of oral microorganisms differ from those found in the postpartum and nonpregnant states^25^, ^26^. Varying physiological and metabolic conditions during pregnancy could explain this difference.

In addition to age, dietary habits play a crucial role in shaping the oral microbiota. Human diets and living habits have undergone significant changes over time. Recent dental calculus studies, including research on fossils from ancient times, have indicated a decrease in the diversity of oral bacteria and an increase in the number of pathogenic bacteria^27^. Differences in diet between groups of modern humans, such as hunter‐gatherers, farmers, and urban residents, result in variations in the diversity and richness of the oral microbiota. Furthermore, the oral microbiota of hunter‐gatherers appears to be more like those of ancient populations, likely due to their lifestyle and dietary habits^28^. Understanding the impact of age and diet on the oral microbiota is essential for developing effective interventions and treatment strategies to maintain oral health.

In addition, oral hygiene habits may affect the oral microbiota, with scraping having the most significant effect on the microbiota^29^. Scraping destroys biofilms, diminishes oral microbiome diversity, and promotes biofilm rebuilding. Ethnicity, geography, and many other factors contribute to differences in diet, resulting in the development of unique oral microbiota.

## INFLUENCES ON THE FORMATION OF THE ORAL MICROBIOME

The composition and stability of oral microbiota are affected by many factors, including salivary flow, chemical substances, bacterial interactions, pathogenic infections, and the host's immune status.

### Salivary flow and external forces

The bacterial colonization of the oral cavity is heavily influenced by the oral matrix—particularly by the renewal of saliva and shedding of the matrix^10^, ^30^. A person's salivary flow is constantly changing, which can affect the adherence of bacteria to solid surfaces in the mouth and the distribution of substances in the oral cavity. In turn, this shapes the structure of the bacterial community. Reduced salivary flow, which occurs during sleep, promotes the proliferation of bacteria, including *Lysobacter*‐type species, *S. salivarius*, *P. melaninogenica*, *P. veroralis*, and *P. pallens* ^31^. The loss of the oral matrix can affect bacterial colonization and biofilm renewal, especially in the hard palate and other areas of the oral cavity. The bacterial community attached to the tooth surface is the most stable because of the tooth's solid, constant tissue. By contrast, while mucosal cells renew rapidly, the bacterial community in the mucosal epithelium changes the most frequently^32^.

External forces, such as chewing gum^33^, brushing^34^, cleaning, washing^35^, and scraping, can interfere with the oral microbiota. Among these factors, scraping has the most significant effect on microbiota^29^. In the study, before scraping, the bacterial diversity in the dental plaque was significantly higher than that in the saliva. This diversity decreased after scraping and reached its lowest level on the third day. After the biofilm was destroyed, many bacteria from the saliva migrated into the dental plaque. The biofilm began to rebuild after the third day, and the diversity of the bacterial species increased. After 3 months, the biofilm recovered. Researchers have also investigated other methods of treating periodontitis, such as ozonized water irrigation and mechanical debridement, which have proven to be more effective for irrigation than normal saline^36^.

### Chemical substances

Oral gases and salivary components play fundamental roles in the distribution and growth of bacteria in the oral cavity. Oral gases, predominantly oxygen, create an environment that hinders the growth of anaerobic bacteria on the surface of the microbiota, resulting in increased bacterial growth in the gingival sulcus and other areas. Saliva is primarily composed of water but also contains a range of nutrients, such as sugars, polypeptide proteins, and vitamins, that can provide nourishment and promote bacterial growth^37^.

Saliva contains inorganic ions, such as Na^+^, K^+^, Cl^−^, and PO_4_^3−^, whereas organic acids are mainly derived from bacterial metabolism. Small amounts of specific ions are essential for the growth of specific bacteria; a lack of these ions inhibits bacterial growth, affecting the entire structure of the bacterial community. For example, black‐pigmented anaerobes require ferrous ions from host hemoglobin. Bacteria that contain nitroreductase decompose nitrate derived from dietary intake^38^, leading to the formation of nitrite^39^, which can inhibit the growth of certain acidophilic bacteria^40^. Recent studies have shown that increasing dietary nitrate can effectively inhibit dental caries^41^, ^42^. These findings indicate that altering the composition of salivary microbiota can have a targeted prevention and treatment effect on diseases^43^, ^44^. Meanwhile, nanosilver and nanocalcium hydroxide can disrupt the bacterial biofilm^45^.

In addition to nutrients, non‐nutritive sweeteners can affect the growth of oral bacteria. Saccharin reduces the abundance of *Fusobacterium*, whereas aspartame diminishes *Porphyromonas* and *Prevotella* ^46^.

Using a mouthwash that contains enzymes can reduce the burden of dental microbiota in patients with fixed orthodontic appliances without affecting their salivary microbial composition^47^. Some mouthwashes that contain special chemicals, such as cetylpyridinium chloride and O‐cymen‐5‐ol, can also achieve specific antibacterial effects to prevent dental problems^48^.

### Host innate immunity

Each host releases various peptides and proteins, such as antimicrobial peptides, defensins, secretory Immunoglobulin A (IgA), and other substances^49^, into the saliva to regulate the oral microbiota. Antimicrobial peptides, histidine‐rich polypeptides (HRPs), and defensins can inhibit various organisms, including bacteria, fungi, and viruses^50^. In vitro experiments have shown that the bactericidal effect of defensin depends on the concentration of saliva and serum diluted in the buffer. Secretory IgA attaches to specific bacteria and mediates their adhesion to oral epithelial cell surfaces to form biofilms^51^, ^52^, ^53^.

Individuals infected with the human immunodeficiency virus (HIV) experience damage to their immune systems, causing a decline in immune function that makes it difficult to fight pathogenic bacteria. Consequently, a wide variety of pathogenic bacteria can rapidly colonize the oral cavity, leading to a high incidence of oral candidiasis in patients with acquired deficiency syndrome (AIDS)^54^.

### Bacterial interactions

Bacteria form symbiotic relationships through adhesin recognition and glycan binding. Certain receptors on the surface of *Streptococcus* contain galactose and n‐acetylgalactose motifs that resemble those in the host, allowing them to recognize and bind to other bacteria^55^, ^56^. Therefore, *Streptococcus* often serves as the foundation for assembling the microbial community. In one study, fluorescence labeling revealed that the bacterial community adopted a corncob‐like structure, with aerobic bacteria primarily situated on the outermost layer and anaerobic bacteria predominantly located in the deeper layers^32^. *Streptococcus*, a facultative anaerobic bacterium, has been found to produce lactic acid, acetic acid, and hydrogen peroxide internally and externally. These byproducts promote the aerobic respiration of *Corynebacterium* and encourage the formation of long filaments on the tooth surface. The “corncob” structure stabilizes the bacterial community, anchoring it firmly. *Po. gingivalis* produces p‐aminobenzoic acid, which acts as a signaling molecule by binding to *Streptococcus* cell surface receptors. This interaction modulates downstream signaling pathways and specific enzyme activities, ultimately influencing cell metabolism^57^.

In addition to symbiotic relationships, bacteria sometimes exhibit competition and mutual inhibition. *Streptococcus*, which dominates the oral cavity, produces a polypeptide called bacteriocin, which acts as an antibiotic and inhibits the growth of other bacteria, such as *F. nucleatum*, *Po. gingivalis*, *Scardovia wiggsiae*, *Dialister invisus*, *Actinomyces* sp., *Capnocytophaga leadbetteri*, *Corynebacterium matruchotti*, *P. denticola*, *F. nucleatum*, and *Atopobium parvulum* ^58^. Bacteriocin is a potent molecule that enables *Streptococcus* to maintain its dominance in the oral microbiota^13^.

Bacterial communities often exhibit synchronous responses to extracellular signaling molecules, resulting in uniform changes in their behavior. This phenomenon, known as quorum sensing^59^, also occurs in the oral microbiota^60^. Quorum‐sensing signaling molecules encompass a variety of peptides, enzymes, and esters, including acyl‐serine lactones and oligopeptides^61^. Studying quorum sensing and its signaling molecules helps us understand bacterial interactions and identify potential strategies for modulating human microbiota^62^.

Administering probiotics, such as *Bifidobacterium animalis* subsp. *lactis* BL‐11, has effectively enhanced oral microbial diversity in children with Prader–Willi syndrome^63^. This led to an increase in *Faecalibacterium*, *Paracoccus*, and *Leptotrichia*, improving metabolic function, promoting growth and cognitive development, and enhancing social behavior^64^. Unlike mouthwashes that contain chlorhexidine or sodium fluoride, probiotic mouth rinses demonstrate no significant capacity for treating *Streptococcus mutans*, but they can effectively inhibit the proliferation of pathogenic bacteria^65^. Moreover, probiotic mouthwash was observed to help control periodontitis after mechanical therapy better than a placebo mouthwash^66^, ^67^.

### Viral infection

Several microorganisms can infect the oral cavity, influencing the diversity and abundance of oral bacteria. In a study of the dynamic changes in oropharyngeal bacteria in healthy individuals infected with the influenza virus, no statistically significant differences in most bacteria were found between the infected and control groups^68^. However, a few bacteria, such as *Prevotella*, exhibited a decrease in proportion that was reversible within 1 month of infection. This indicates that oropharyngeal microbiota can adapt flexibly to influenza infection, with little impact from the viral invasion.

Infectious pathogens can alter the structure, quantity, and migration of oral microbiota. In the case of respiratory viral infections, such as severe acute respiratory syndrome, influenza, and COVID‐19, oral bacteria have been found to colonize the lungs. Certain oral bacteria, including opportunistic pathogens such as *Candida* and *Pseudomonas*, can migrate to the lungs and cause local infections^69^.

### Pathological conditions

Many diseases can affect the oral microbiome, including diabetes, autoimmune diseases, and psychological distress. Diabetes increases cytokine expression and inflammation. In addition, diabetes affects both the innate and adaptive immune responses, contributing to periodontitis. A decrease in the number of neutrophils alters the biofilm in the gingival crevice^70^. Moreover, bacteria can take advantage of high levels of glucose in the environment, leading to chronic inflammation in the periodontal tissues^71^. Sjögren's syndrome is a chronic systemic autoimmune disease that decreases the salivary flow rate and changes salivary constituents. The composition of the oral microbiome varies with changes in interferons, lymphocytes, and antigen presentation^72^. Finally, chronic psychological distress suppresses the diurnal secretion of salivary glucocorticoids and catecholamines, which regulate gut microbes and thus attenuate diurnal rhythms and functional microbial pathways^73^.

## ORAL MICROBIOME AND HUMAN DISEASES

Local disorders in the oral microbiome may lead to diseases within the oral cavity, such as dental caries, periodontitis, and oral cancer. Moreover, bacteria that colonize the oral cavity can migrate to other parts of the body and trigger infectious or autoimmune diseases resulting from the body's response to the infection; some bacteria can also induce or exacerbate digestive tract cancers in distant organs^8^. Oral microbial characteristics and pathogeneses in patients with different diseases are summarized in Table 1.

**Table 1:** Diseases associated with the oral microbiome.

System	Diseases	Main species	Biological functions	References
**Oral cavity**	Dental caries	*Streptococcus mutans*, *Lactobacillus* spp., *Bifidobacterium dentium*	Produce organic acids from fermentable carbohydrates that demineralize tooth tissues.	[74, 75, 76]
	Periodontitis	*Porphyromonas gingivalis*, *Treponema denticola*, *Tannerella forsythia*	Activate receptors TLR1–TLR2 and arginine‐specific gingivalis, inhibit antibacterial response and phagocytosis.	[77, 78, 79, 80]
	Oral cancer	*Fusobacterium nucleatum*, *Clostridium periodontium*, *Prevotella* sp., *Pseudomonas aeruginosa*, *Streptococcus* spp.	Produce proteases to degrade host tissues, break down physical barriers, and affect immune responses. Produce lactic acid to reduce the pH of the oral cavity, which contributes to cancer.	[21, 81, 82, 83, 84, 85, 86, 87, 88]
**Respiratory system**	Respiratory system infection	—	Viral and bacterial coinfection.	[89, 90, 91, 92, 93, 94]
	Cystic fibrosis	*Candida albicans*	Oral bacteria migrate to the lung and cause infection.	[95]
	Allergic asthma	*Filifactor alocis*	Microbial diversity changes, leading to host immunity alterations.	[96]
	Pediatric obstructive sleep apnea	*Firmicutes, Proteobacteria, Bacteroidetes, Fusobacteria*, and *Actinobacteria*	Bacterial alterations cause metabolic disorders in the host.	[97]
**Digestive system**	Gastric cancer	*Slackia*, *Selenomonas*, *Bergeyella*, *Capnocytophaga*, and *Neisseria*	These oral bacteria continuously increase from superficial gastritis to gastric cancer.	[98, 99, 100, 101, 102]
	Esophageal squamous cell carcinoma	*Lautropia*, *Bulleidia*, *Catonella*, *Corynebacterium*, *Moryella*, *Peptococcus*, and *Cardiobacterium*; *Prevotella*, *Streptococcus*, and *Porphyromonas*	Consuming more pickled vegetables and brushing teeth less often increase pathogens, which increase nitrites, leading to esophageal cancer.	[103, 104, 105]
	Colorectal cancer	*Fusobacterium nucleatum* and *Filifactor alocis*	Produce Fap2 to recognize Gal–Gal–NAc, which binds to colorectal cancer to promote its formation; bind natural killer cells to inhibit their cytotoxicity.	[106, 107]
	Pancreatic cancer	*Neisseria elongata*, *Streptococcus mitis*, *Porphyromonas gingivalis*, and *Aggregatibacter actinomycetemcomitans*; *Granulicatella adiacens* and *Leptotrichia* sp.	*Neisseria longifolia* and *Streptococcus mitis* decrease, and adjacent granular bacteria increase. Induce autoimmune diseases and pancreatic inflammation to develop into pancreatic cancer.	[108, 109]
**Nervous system**	Alzheimer's disease	*Porphyromonas gingivalis*, *Treponema denticola*, and *Tannerella forsythia*	The BBB, OMVs and LPS cause neuronal degeneration and tau protein hyperphosphorylation. The release of porin‐like proteins increases the membrane permeability of neurons, causing calcium leakage and impairing nerve conduction.	[110, 111, 112]
**Endocrine system**	Thyroid hormone production increases	*Firmicutes* and *Bacteroidetes*	Bacterial metabolites affect human metabolism.	—
	Diabetes	—	Antiperiodontitis cytokines induce prediabetic diseases; treating diabetes can improve periodontitis.	[113, 114, 115, 116, 117, 118, 119]
**Cardiovascular system**	Rheumatic heart disease	*Porphyromonas gingivalis*, *Streptococcus* spp., and *Hemophilus* sp.	The cross‐reacting antigen leads to autoimmune damage.	[119, 120, 121]
	Ischemic heart disease, peripheral artery disease, atrial fibrillation	*Streptococcus mutans*	*Streptococcus mutans* enters the circulation, promoting the development of atherosclerotic plaques.	[121]
**Skeletal system**	Rheumatoid arthritis	*Porphyromonas gingivalis*, *Streptococcus* spp., and *Hemophilus* sp.	Cross‐reacting antigen leads to autoimmune damage; producing peptide‐arginine deaminase disrupts intracellular signal transmission.	[122, 123, 124, 125, 126, 127]

BBB, blood–brain barrier; LPS, lipopolysaccharide; OMVs, outer membrane vesicles; TLR, Toll‐like receptor.

Bacteria can affect the host's overall health and trigger diseases in multiple organs. Oral pathogens can migrate and cause damage to distant tissues, including the respiratory and digestive tracts, and can even cross the blood–brain barrier, leading to various infections and increasing the risk of Alzheimer's disease^89^, ^110^. Bacterial metabolites can also damage tissues, leading to dental caries and digestive tract cancers^74^, ^120^. Bacteria, particularly *Streptococcus* spp., can induce the production of specific cross‐antibodies that trigger autoimmune diseases, such as rheumatoid arthritis (RA), rheumatic heart disease, pancreatic damage, and diabetes^122^.

### Oral diseases

Oral diseases such as dental caries, periodontal diseases, mucosal diseases, and oral cancer have been linked to changes in the oral microbiome^128^.

#### Dental caries

Under health conditions, an ecological homeostasis between the activity and combinations of microbes maintains the biofilm's health and stability. However, a biofilm can provide a refuge for pathogens and cause them to infiltrate the host. Therefore, to avoid the development of disease, biofilms should be removed^129^. Dental caries is a classic biofilm‐induced disease characterized by the formation of cariogenic biofilms in response to certain host diets^130^.

Inadequate oral hygiene practices can lead to the accumulation of food particles, particularly carbohydrates, which attract acidophilic bacteria, such as *S. mutans*, that produce acidic substances, corrode the tooth surface, and demineralize the tooth's calcium. By penetrating the dentin, continued demineralization can cause the tooth to become fragile and form cavities, disintegrating and destroying tissues. The prolonged presence of acidophilic bacteria leads to a continuous decrease in the pH of the tooth surface and the accumulation of acidophilic bacteria. Effective cleaning is necessary to prevent or arrest this process^74^, ^75^. Interestingly, some patients with caries have low levels of *S. mutans* but high levels of *Lactobacillus* spp. and *Bifidobacterium dentium* ^76^, which can also produce acid and cause caries. This phenomenon is attributed to the ability of *Streptococcus* spp. to produce NO, which inhibits the growth of other pathogens. Additionally, certain strains of *Streptococcus*, such as *Streptococcus dentisani* and *S. oralisis*, are considered probiotics that can produce bacteriocin to inhibit the growth of *S. mutans* ^131^. Regular brushing with fluoride toothpaste and the incorporation of nitrates into one's diet, which transforms the bacteria into NO, can mitigate the growth of acidophilic bacteria and prevent dental caries^132^.

#### Periodontal diseases

Five major microbial complexes (red, orange, yellow, green, and purple) have been found to be involved in the development of periodontal diseases ranging from gingivitis to periodontitis, with the orange and red complexes most often implicated. The orange complex contains a high frequency of *Eubacterium nodatum*, *Parvimonas micra*, and *S. constellatus*, which contribute to endodontic‐periodontal lesions, while the red complex, which contains a high frequency of *Po. gingivalis*, *Treponema forsythia*, and *T. denticola*, is associated with periodontitis severity^133^. Changes in the composition of dental biofilms induce gingivitis, and untreated inflammation perpetuates these compositional changes, resulting in periodontitis^134^.

These pathogens are associated with periodontitis because of their pro‐inflammatory effects. *S. constellatus* produces detoxification enzymes, which allow reactive oxygen species to evade the immune system, and hydrogen sulfide, which increases resistance to cell lysis by the immune system. *Po. gingivalis* activates Toll‐like receptors 1 and 2 (TLR1/TLR2) in host cells, leading to the ubiquitination and proteasomal degradation of the downstream myeloid differentiation primary response gene 88 (MYD88) protein, which inhibits the antibacterial response of host cells. It also produces gingipains, which cause significant damage to periodontal tissues. Additionally, it can activate arginine‐specific gingival sinuses, impede host cell actin aggregation, inhibit phagocytosis, and stimulate the production of inflammatory factors, thereby facilitating the development of periodontitis.

The advent of metagenomics has shed light on the bacterial compositions associated with periodontitis. Different bacteria are involved in each stage from gingivitis to cementum loss, generating a gradual change in the bacterial community's structure^77^. Herpes virus infections may induce immunosuppression and bacterial overgrowth, triggering periodontitis^78^. The numbers of oral *Streptococcus* and *Enterococcus* in mice increased during periodontitis development, whereas those of *Escherichia coli*, *Lactobacillus*, and *Propionibacteria* decreased^79^. Human experiments have demonstrated that even a low concentration of *Po. gingivalis* can change the oral bacterial biofilm, leading to an increase in *Spirochetes*, *Synergistetes*, *Prevotella*, *Fusobacterium*, and *Firmicutes* ^80^.

#### Oral cancer

Oral squamous cell carcinoma is the most prevalent oral cancer, accounting for more than 50% of all cases, yet it exhibits no apparent symptoms during the early stages^81^. Patients with oral cancer have considerably greater bacterial diversity in their oral cavities than healthy individuals^82^. Multiple Gram‐negative anaerobic bacteria, such as *F. nucleatum*, *Clostridium periodontium*, *Prevotella* sp., and *Pseudomonas aeruginosa*, are found at higher levels in the mouths of patients with squamous cell carcinoma^83^. Conversely, patients with squamous cell carcinoma exhibit significantly lower levels of *Streptococcus*, *Veillonella*, and *Rothia* than healthy individuals. The overall abundance of *Streptococcus* spp. decreases with cancer progression^84^. Conversely, an increase in some species considered cancer‐related pathogens, such as *S. anginosus*, *S. constellatus*, *S. salivarius*, *S. gordonii*, and *S. parasanguinis*, has been observed. However, whether the number of *S. mitis* is increasing^85^ or decreasing^86^ in the mouths of oral cancer patients has not yet been clarified.

Alterations in oral pathogens may contribute to the development of cancer through excessive inflammatory reactions and immunosuppression in the host, as well as the induction of malignant transformations, promotion of antiapoptotic activity, and production of carcinogenic substances^87^. Proteases produced by pathogenic bacteria can degrade tissues, destroy physical barriers, alter immune responses, and ultimately contribute to the onset and progression of a tumor in the host. For example, arginine deiminase is considered a potential antitumor drug^88^. The changes in the numbers of *Streptococcus* spp. during the development of cancer and their functions are not clear, probably because *Streptococcus* can produce a variety of metabolites; some can promote cancer, whereas others can inhibit it. For example, lactic acid has a bidirectional effect; it reduces the pH of the oral cavity and contributes to cancer growth. However, it also promotes apoptosis, increases the number of T cells, induces cytokines such as IFN‐γ and TNF‐α, and improves tumor suppression gene expression^21^.

### Nonoral diseases

The imbalance and migration of oral microbiota can result in a range of systemic diseases, which may also impact the oral microbiome to some extent^135^.

#### Respiratory system diseases

The oral and respiratory tracts are interconnected, allowing oral bacteria to migrate and potentially affect the respiratory system, including the lungs^89^, ^90^, ^91^. A recent study showed that decreased lung function and inflammatory response in humans are associated with the accumulation of oral microbiota in the lungs^92^. Coinfections with bacteria are common in COVID‐19 and other respiratory infections, such as influenza^93^. A case report found that *Filifactor alocis* caused extraoral infections^94^. The oral microbiota, particularly *Candida albicans*, has been linked to lung infections in individuals with cystic fibrosis^95^. Changes in the oral microbiota have also been observed in noninfectious respiratory diseases, such as allergic asthma^96^.

Pediatric obstructive sleep apnea can disrupt host metabolites, leading to changes in oral microbiota, particularly *Firmicutes*, *Proteobacteria*, *Bacteroidetes*, *Fusobacteria*, and *Actinobacteria*, and potentially increasing the risk of dental caries^97^.

#### Digestive system **diseases**

Dysbiosis of the oral microbiota has been linked to many digestive system diseases, including cirrhosis, gastrointestinal inflammation, and gastric cancer. More than half of the bacteria associated with cirrhosis enter the body through the mouth, and oral bacteria can easily be transferred to the gastrointestinal tract and cause inflammation. Maladjustment of the oral microbiota increases the risk of gastric cancer after the onset of periodontal disease^98^. Additionally, the oral cavity is a potential reservoir of *Helicobacter pylori* ^99^. Researchers have developed a system for scoring oral microbiome based on the characteristics of the oral microbiome to screen potential patients for potential gastric cancer^100^.

Because of variations in age, sex, and race, there are currently no definitive conclusions about the changes in the microbiome that occur as gastritis progresses to gastric cancer. However, research has indicated an increase in the abundance of four genera (*Slackia*, *Selenomonas*, *Bergeyella*, and *Capnocytophaga*), which are primarily found in the oral microbiome, during the development of superficial gastritis, atrophic gastritis, gastric intraepithelial neoplasia, and ultimately gastric cancer^101^, ^102^.

In one study^103^, the overall diversity of the oral microbiome in patients with esophageal squamous cell carcinoma (ESCC) was significantly lower than that in patients with dysplasia and healthy controls. Moreover, several genera, including *Lautropia*, *Bulleidia*, *Catonella*, *Corynebacterium*, *Moryella*, *Peptococcus*, and *Cardiobacterium*, were less abundant in patients with ESCC, whereas *Prevotella*, *Streptococcus*, and *Porphyromonas* were more abundant. These findings suggest a clear association between the oral microbiota and the risk of developing ESCC. In addition, patients with ESCC were found to consume more pickled vegetables than those with dysplasia and healthy controls, and both patients with ESCC and dysplasia brushed their teeth less frequently than healthy controls^103^. These lifestyle factors may contribute to changes in saliva composition and pH levels, leading to differences in the oral microbiome and the development of ESCC^104^, ^105^.

*F. nucleatum*, which is common in humans but rare in healthy guts, is associated with colorectal cancer. It can attach to colorectal cancer cells and promote colonic tumor formation. It produces Fap2 to recognize and bind Gal‐Gal‐NAc, which is overexpressed in colorectal cancer^106^. After localization to the tumor, Fap2 binds to natural killer (NK) cells and inhibits their cytotoxicity^107^.

Another study^108^ compared patients with pancreatic cancer, pancreatic inflammation, and controlled pancreatitis to healthy individuals. This study revealed notable variations in oral bacteria across these groups, with the greatest differences observed between patients with cancer and healthy individuals. Specifically, *Neisseria elongata*, *S. mitis*, *Po. gingivalis*, and *A. actinomycetemcomitans* were significantly reduced in patients with pancreatic cancer, whereas *Granulicatella adiacens* and *Leptotrichia* spp. increased notably, indicating a potential association with pancreatic cancer^108^. Significantly increased numbers of bacteria have been found to induce autoimmune diseases and multiple types of inflammation, leading to pancreatic cancer^109^. Patients with controlled pancreatitis exhibited an improvement in their oral microbiota. However, it remains unclear whether this improvement helps alleviate the disease or, contrarily, whether disease remission leads to the recovery of the microbiome.

#### Nervous system diseases

In patients with Alzheimer's, the levels of oral anaerobic bacteria in the brain, such as *T. denticola*, *T. forsythia*, and *Po. gingivalis*, are significantly higher than in healthy individuals^110^. Studies have suggested that these bacteria can penetrate the blood‐brain barrier and secrete outer membrane vesicles (OMVs) and lipopolysaccharides (LPSs), leading to the activation of glial cells, nerve inflammation, the degeneration of neurons, the phosphorylation of tau protein, and ultimately cell death. Furthermore, these bacteria produce porin‐like proteins that increase the membrane permeability of neurons, disrupt calcium levels, and impair nerve function^111^. Evidence suggests that regular oral health interventions, including consistent tooth brushing and the use of mouthwash with chlorhexidine, can help modify the subgingival microbiota and potentially slow cognitive decline in individuals with Alzheimer's disease^112^.

#### Endocrine system diseases

Changes in the oral microbiome can cause hormonal imbalances, such as insulin resistance, affect glucose metabolism and carbohydrate levels in saliva, and exacerbate oral diseases^136^, ^137^. Sialic acid, which can disrupt the oral microbiota and increase the risk of dental erosion, caries, and gingivitis, is also associated with obesity and high blood sugar^113^. There is a clear epidemiological link between diabetes and periodontal disease^114^, ^115^, and the two conditions can influence each other^116^. Treating diabetes with insulin and maintaining long‐term metabolic control can improve gingivitis and reduce gingival redness and swelling^138^. Moreover, periodontitis patients with noninsulin‐dependent diabetes mellitus have higher levels of *Bacteroides intermedius* and *B. gingivalis* in their oral microbiota than those with physiological glucose tolerance^117^. Patients with chronic periodontitis produce a variety of cytokines in response to infections caused by changes in oral microbiota, among which MCP‐1, GM‐CSF, IL‐6, IL‐5, and IFN‐γ are thought to be associated with type 2 diabetes. These cytokines may induce prediabetic diseases, such as autoimmunity, insulin resistance in adipocytes, fat accumulation in macrophages, and some vascular complications^118^. Women with gestational diabetes mellitus also exhibit changes in oral microbes with less diversity; *Selenomonas* and *Bifidobacterium* levels increase, whereas *Fusobacteria* and *Leptotrichia* levels decrease^119^.

#### Cardiovascular diseases

Oral microbiota can convert dietary nitrate ions into NO under specific conditions. When NO diffuses into the bloodstream, it dilates blood vessels, reduces peripheral blood flow resistance, lowers blood pressure, and increases cardiac output, thereby alleviating hypertension in older individuals to some extent. However, the effect on healthy young people is not significant^120^. In contrast, patients with caries, gingivitis, and periodontitis are more likely to develop structural deficiencies and functional abnormalities of the heart valves owing to oral and systemic infections^139^. Numerous studies have reported an association between periodontal disease and various heart diseases, including ischemic heart disease, peripheral artery disease, and atrial fibrillation. Oral bacteria play an important role in this link. Many bacteria have cross‐reacting antigens that cause autoimmune damage to periodontal pathogens and the components of the intima wall. Additionally, *S. mutans* in the oral cavity can directly enter circulation, promoting the development and progression of atherosclerotic plaques^121^.

#### Orthopedic disease

*Streptococcus* and *Hemophilus* infections provoke the body to produce antigens that cause RA, particularly at mucosal sites in the mouth^122^, ^123^. The level of serum anti‐LPS from *Po. gingivalis* Immunoglobulin G (IgG) antibodies is also associated with RA^124^. In addition, oral *Po. gingivalis* can produce peptide‐arginine deaminase^125^, ^126^, which converts arginine into citrulline. This alteration in the amino acid sequence of some human proteins can affect their overall structure and disrupt intracellular signal transmission, ultimately affecting the production of immune factors^127^. Consequently, patients with RA are more likely to develop periodontitis than those with osteoarthritis. Fortunately, the dysregulation of the oral microbiota associated with RA can significantly improve in patients treated with disease‐modifying antirheumatic drugs^140^.

## POSSIBLE MECHANISMS OF ORAL BACTERIAL INVOLVEMENT IN DISEASES

**Figure 2:**
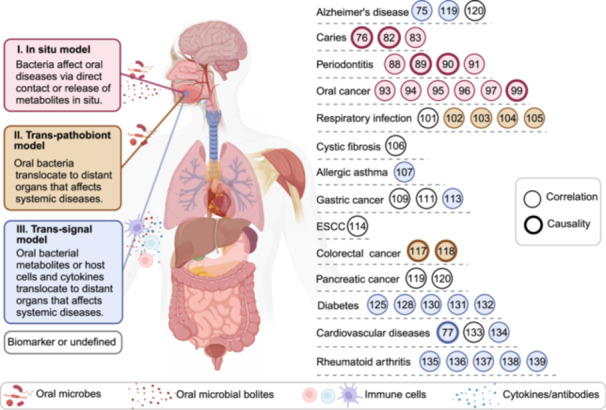
Specific mechanisms through which oral microbiota influence disease. Bacteria can directly damage oral and distant tissues, leading to oral and distant organ diseases. These diseases occur and progress via three mechanisms (in situ model, trans‐pathobiont model and trans‐signal model). In every category, we provide some examples of diseases with their corresponding pathogens, pathogenic mechanisms, and references (in circle). Illustrations created with BioRender.com.

We have summarized the current literature and proposed that oral bacteria principally contribute to the occurrence and progression of diseases via the following three mechanisms (Figure 2).

### In situ model

An increase in oral pathogens can lead to in situ diseases. For example, *Po. gingivalis* can cause direct damage to oral tissues, leading to periodontitis^80^. Additionally, the presence or absence of biofilm formation or the increased virulence of pathogenic bacteria may lead to oral diseases. *S. mutans* can also cause caries^74^, ^75^ through its metabolites, such as acidic substances. Metabolites corrode the tooth surface and demineralize the tooth's calcium. In addition, lactic acid reduces the pH of the oral cavity and contributes to oral tumor growth. However, the causal relationship between oral cancer and the oral microbiome remains unclear. Lactic acid can also promote apoptosis, increase the number of T cells, induce inflammatory cytokines, and increase the expression of tumor suppression genes^21^.

### Trans‐pathobiont model

The oral, digestive, and respiratory tracts are interconnected, which allows oral bacteria to migrate and potentially affect other organs, including the respiratory system^89^, ^90^, ^91^ and the digestive tract^106^. Pathogen migration may decrease organ functionality and enrichment and lead to an inflammatory response in the lungs^92^. Pathogens can also migrate and damage distant tissues, leading to cancer. For example, *F. nucleatum* can translocate to the colorectum and help tumor cells escape NK cell cytotoxicity^107^.

### Trans‐signal model

Bacterial infections can affect the host's metabolism, cell signaling pathways, and immune functions, contributing to diseases. *Streptococcus* spp. infection can cause the body to produce specific cross‐antibodies, triggering autoimmune diseases such as RA^123^, ^124^ and rheumatic heart disease^121^, ^139^. Pathogens associated with periodontitis disrupt the immune barrier, modify signal transduction pathways, and affect the secretion of immune factors. Evidence suggests that periodontitis can lead to immune system dysfunction and increase the incidence of other diseases, such as chronic obstructive pulmonary disease^141^, gastric cancer, pancreatic cancer^116^, and diabetes^77^, ^98^. Additionally, periodontitis may increase the host's susceptibility to various maternal diseases and raise the odds of the transmission of HIV from the amniotic fluid or vagina to infants^142^.

Bacterial metabolites can also be transferred throughout the body and cause various diseases, such as esophageal cancer^103^ and pancreatic cancer^109^. *Po. gingivalis* secretes OMVs and LPSs, which cross the blood–brain barrier and phosphorylate tau proteins, leading to a degeneration of neurons^111^. In contrast, NO has potential benefits, such as promoting muscle activity and lowering blood pressure^74^, ^120^.

Many bacteria have an unclear causal relationship with diseases and can only be used as biomarkers with quantitative relationships, such as *Slackia*, *Selenomonas*, *Bergeyella*, and *Capnocytophaga* in gastric cancer^101^, ^102^; *Prevotella*, *Streptococcus*, and *Porphyromonas* in ESCC^104^, ^105^; and *G. adiacens* and *Leptotrichia* spp. in pancreatic cancer^108^. Further studies are needed to gain insight into the specific roles of bacteria in disease development.

## HETEROGENEITY OF STUDIES AND CAUSALITY DETERMINATION

The association between bacteria and diseases is often primarily based on quantity; however, the specific mechanisms remain unclear. Quantitative relationships can vary or exhibit contradictions between different studies. This could be attributed to the heterogeneity of the experimental samples and bacterial strains, which may have influenced the outcomes. Furthermore, different species within the same genus, or even the same species, may have varying effects on the same disease. Consequently, conducting further experiments with minimal heterogeneity to determine causality and investigate the interactions between the oral microbiome and diseases is crucial.

### Variation in samples and individuals

The heterogeneity of existing experimental samples can be primarily attributed to the limitations of the experimental methods and designs employed. Factors such as diet, race, age, and lifestyle contribute to this heterogeneity. When the number and sources of samples are limited, it is crucial to address heterogeneity by increasing the sample size. Additionally, including samples from diverse sources is advised, as studies have reported contradictory conclusions regarding the quantitative relationship between, for instance, *S. mitis* and oral cancer^85^, ^86^.

Furthermore, researchers must establish consistent baselines for health conditions and control for other influencing factors. As previously mentioned, the compositions of oral bacteria vary across locations. Therefore, when collecting samples, multiple sites should be considered.

### Variation in microbes

**Figure 3:**
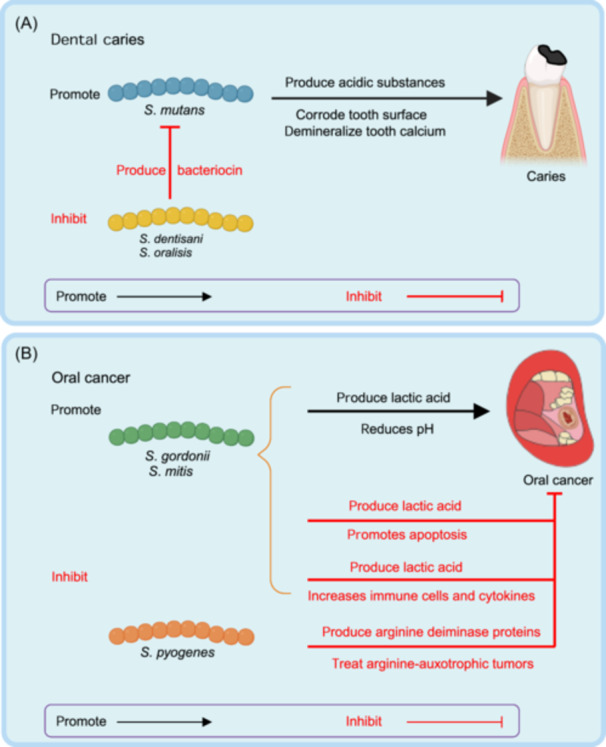
Different *Streptococcus* spp. have distinct effects on the same disease through metabolites. (A) Different species of *Streptococcus* have different effects on dental caries. *S. mutans* produces acidic substances that corrode the tooth surface, demineralize tooth calcium, and cause caries^74^, ^75^; by contrast, *S*. *dentisani* and *S*. *oralisis* produce bacteriocin to inhibit the growth of *S. mutans* and relieve caries^131^. (B) Different *Streptococcus* spp. have different effects on oral cancer. *S*. *gordonii* and *S*. *mitis* produce lactic acid that reduces the pH of the oral cavity, contributing to cancer; however, lactic acid can also promote apoptosis and increase immune cells and cytokines that suppress tumors. *S. pyogenes* produces arginine deiminase to deplete arginine and inhibit tumor growth^21^. Illustrations created with BioRender.com.

The heterogeneity of oral microbes at the strain level also has significant implications. For example, different *Streptococcus* species exert varying effects on the same disease. *S*. *mutans* is known to produce acidic substances and contribute to the development of caries^74^, ^75^, whereas *S*. *dentisani* and *S*. *oralisis* are probiotics that produce bacteriocins, which inhibit the growth of *S. mutans* and alleviate caries^131^ (Figure 3A). Similarly, the same species can have different effects on oral cancer. For example, the lactic acid produced by *S*. *gordonii* and *S*. *mitis* lowers the pH of the oral cavity and contributes to cancer development; however, it also promotes apoptosis and increases the number of immune cells and cytokines that suppress tumors^21^ (Figure 3B). In the clash between promoting and inhibiting effects, the dominant factor will ultimately determine the outcome.

Compared with the gut microbiome, which has been widely studied, there is a paucity of research on the oral microbiome. Additionally, it is important to consider the spatial and temporal universality of the oral microbiome, as long‐term cohort studies and meta‐analyses are lacking in this area. To gain a better understanding of the role of oral microbiome in health, further experimental studies that use innovative tools and follow robust guidelines are needed^143^, ^144^.

### Research strategies from the species level to the strain level

Analyses focused on the strain level are of great significance for the study of the oral microbiome. However, recent studies have primarily focused on the number of microbes. Diverse species within genera add to functional heterogeneity, leading to conflicting conclusions. To improve the accuracy of such research, the functional diversity of strains and individual strains must be considered. This requires innovative research models because relying solely on sequencing‐based methods is insufficient. At the strain level, researchers can examine the complexity and variation in microbe behavior and influence, which cannot usually be captured by a broader taxonomic analysis.

The widespread use of culture‐free methods helps mine microbial functions and species abundance, aiding the discovery of disease‐related molecules. However, current analysis pipelines, such as Kraken^145^ and Metaphlan^146^, suffer from false positives and sensitivity issues. The lack of an oral microbiome database makes the interpretation of these results challenging. Sample collection, storage, and processing biases exacerbate this issue. In addition, several pipelines are emerging, including PanPhlAn^147^, StrainPhlAn^148^, and inStrain^149^, that can trawl signatures to reveal microbial strains and their functions in the deep sequencing of metagenomic data. The development and application of culturomics provide important information for future strain‐level research.

### Causality determination

**Figure 4:**
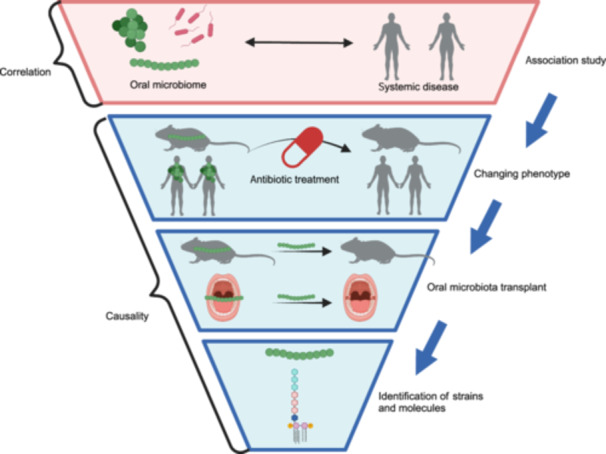
Improving the experimental framework to determine causal relationships. The study framework consists of four levels. First, the bacterial species that have demonstrated a quantitative relationship with a disease are identified by comparing healthy and diseased individuals. Second, antibiotics are used to alter the disease phenotypes displayed by humans or mice compared with untreated or conventional controls. Third, the oral microbiota is transplanted from a donor to a recipient, and phenotype transfer is confirmed. Finally, the specific strains and molecules that elicit the phenotype are identified. Illustrations created with BioRender.com.

Exploring the results of experiments to determine causal links would help solve the problem of heterogeneity among different studies. To establish a precise causal chain, rigorous scientific experiments must first be conducted to identify quantitative relationships. In addition to the diversity of the collected samples, other aspects must be improved. To uncover host phenotypes affected by the oral microbiome, we propose a chain of study frameworks (Figure 4) pertaining to correlation and causality, including (1) association studies, (2) observations of gnotobiotic animals and antibiotic‐treated animals and humans, (3) oral microbiota transplants, and (4) the identification of the strains and molecules that elicit certain phenotypes. Application of the multi‐omics approach to encompass, for instance, the microbiome, metabolome, host transcriptome, and proteome, experimental validation both in vivo and in vitro, and completion of clinical longitudinal or interventional studies will help determine causality beyond correlations.

## PROSPECTS AND APPLICATIONS

A close association between the oral microbiome and human health has been established, and variations in the oral microbiome have been linked to numerous disorders. Therefore, investigating the differences between the oral microbiomes of healthy individuals and patients may facilitate early diagnosis and improve our understanding of disease etiology and pathogenesis. Furthermore, manipulating the oral microbiome of healthy individuals or patients may prevent or treat specific diseases^150^, ^151^, ^152^. However, scientific issues and gaps emerge when studying the association between oral bacteria and diseases. These include deficits in systematic theoretical guidance, research techniques, and data analysis methods—particularly the absence of interventional studies that verify the causal relationships between oral bacteria and diseases. Addressing these gaps in future studies would significantly enhance our ability to prevent and treat various disorders.

### Early discovery and prevention of diseases

The relationship between disorders of the oral microbiome and oral diseases is of considerable importance. Many diseases, such as dental caries, periodontal disease, and oral cancer, are closely related to an imbalance in the oral microbiota. Therefore, preventing and treating oral microbiota imbalances can reduce the risk of these diseases. In addition to daily oral hygiene management, specific measures can help adjust the balance of the oral microbiota, such as eating more probiotic‐rich foods^63^, using oral cleansers with probiotics^65^, using chemical cleansers and mechanical measures^153^, and filtering drinking water. Moreover, controlling habits such as overeating and smoking^154^ is an effective way to prevent oral microbial dysbiosis. In summary, factors such as diet, lifestyle, and medications can affect the balance of the oral microbiome, and adverse effects may contribute to oral diseases. Therefore, the maintenance and balance of oral microbiome are important and can effectively prevent oral health problems.

One way to prevent dental caries is to regularly monitor the composition of a child's oral microbiota and assess their status score. In particular, focusing on the detection of *S. mutans*, the oral bacterial species that differs the most between healthy individuals and those with current diseases, can assist in the early detection of the risk of dental caries^155^. Moreover, Alzheimer's disease can be detected early by measuring the number of antibodies against periodontal disease‐related bacteria in the brain. Where increased antibodies are observed, bacteria are more likely to have migrated to the patient's brain, leading to diseases^156^. Therefore, measures should be taken to protect the oral microbiome and restrain the growth and migration of pathogenic bacteria to prevent diseases.^157^

### Treatment of diseases

The oral microbiota is a complex system with diverse regulatory factors. Many methods can be used to inhibit the growth of pathogenic bacteria and treat diseases, such as adjusting the chemical composition of the oral cavity. Increasing the amount of high‐nitrate substances in food and producing NO can inhibit the growth of *Acidophilus* and improve dental caries^42^, ^158^. In addition, NO plays a role in lowering blood pressure and relieving hypertension in older adults while regulating mitochondrial respiration, improving muscle movement, and the efficiency of skeletal muscle oxygen use^159^, ^160^.

Symbiotic bacteria can also be used to treat oral diseases. For example, *Lactobacillus reuteri* can be combined with *Candida* species to decrease pathogenic oral microorganisms, improve dental caries, and maintain oral health^161^. In addition, different species of *Lactococcus* have variable acid‐producing abilities that can inhibit the growth of pathogenic bacteria by competing for metabolites, thus regulating the oral microbiota and treating various diseases^162^, ^163^, ^164^, ^165^.

In addition, studies have demonstrated that regulating the oral microbiota can treat food allergies. For example, oral microbial diversity is significantly reduced in people with food allergies, with a significant decrease in certain bacteria leading to increased bacterial metabolites and short‐chain fatty acids that exacerbate allergic reactions^166^. Modulating oral microbiota can increase microbial diversity and thus improve allergy symptoms^167^.

In conclusion, studying the metabolites and regulatory factors of the oral microbiome can help identify other chemicals that may affect human health and discover ways to treat diseases. The maintenance and improvement of human health through intervention in oral microbiome maintenance is a popular research focus^168^.

### Future directions

An imbalanced oral microbiome is associated with various diseases, including periodontitis, which is closely related to the proliferation of pathogenic bacteria. However, the causal relationship between microbial imbalance and diseases remains unclear. It has been demonstrated that certain bacteria in a patient's mouth increase or decrease under particular conditions, but it is unclear whether such imbalances are caused or affected by the diseases in question. These two factors may reinforce each other so that the imbalance exacerbates the development of the disease to some extent, while the disease reciprocally intensifies the imbalance.

Therefore, it is crucial to analyze the causal relationships between diseases and imbalances in the oral microbiome. We need to focus on the specific connections between various diseases and imbalanced oral microbiome as the next important direction of oral microbiota‐related research. We can better prevent and treat these diseases by determining whether an imbalance in the oral microbiome causes the disease or, conversely, the disease leads to an imbalance in the oral microbiota.

In addition to inhibiting the growth of pathogenic bacteria, treatment methods that alter the structure of oral microbiota often limit the growth of beneficial bacteria. For example, scraping the oral microbiota film can destroy the entire oral microbiota ecosystem. Using *S. mutans* to inhibit the growth of pathogenic bacteria also inhibits normal bacterial growth in the oral cavity. Therefore, we need to focus on destroying the growth of pathogenic bacteria while maintaining the integrity of the oral microbiota's structure as much as possible. However, the comprehensive destruction or restoration of the entire oral biofilm may affect the stability and resistance of the oral microbiome. Therefore, other supportive treatment measures should be adopted to reduce the colonization and growth of other pathogens and promote the growth of healthier oral microbiome.
